# Advances in microbial extracellular vesicles: synthetic biology platforms and medical applications

**DOI:** 10.20517/evcna.2025.103

**Published:** 2026-06-22

**Authors:** Zi-Yue-Er Tang, Xin-lu Yang, Yan-Wen Zhou, Hang Zou, Ying Li, Hong Wu, Dai-Xu Wei

**Affiliations:** ^1^Clinical Medical College and Affiliated Hospital of Chengdu University, Chengdu University, Chengdu 610081, Sichuan, China.; ^2^Tianjin Key Laboratory of Conservation and Utilization of Animal Diversity, College of Life Sciences, Tianjin Normal University, Tianjin 300387, China.; ^3^Antibiotic Innovation and Resistance Control Key Laboratory of Sichuan Province, Chengdu University, Chengdu 610081, Sichuan, China.; ^4^The Affiliated Hospital of Southwest Medical University; National Key clinical Construction Specialty, Luzhou 646000, Sichuan, China.; ^#^These authors contributed equally to this work.

**Keywords:** Extracellular vesicles, synthetic biology, bacterial extracellular vesicles, production and separation

## Abstract

Extracellular vesicles (EVs), typically ranging from 20 to 400 nanometers in diameter, are membrane-bound structures released into the extracellular environment by bacteria via specific secretion mechanisms. Consequently, these vesicles play a crucial role in bacterial physiological regulation and communication with hosts. Compared with EVs derived from plants and animals, Microbial extracellular vesicles (MEVs) offer distinct advantages, including lower production costs, higher yields, and greater abundance. This review outlines the biogenesis and release mechanisms of MEVs, and highlights how synthetic biology tools and platforms can be leveraged to engineer these vesicles, such as enhancing their production, modifying their cargo, and tailoring their surface properties. Furthermore, this article examines the promising biomedical applications of engineered MEVs, including targeted drug delivery, immune and inflammatory modulation, the discovery of disease biomarkers and therapeutic development. However, clinical translation of MEVs faces considerable challenges, primarily due to the lack of standardized, universally applicable isolation and purification protocols. This review therefore summarises contemporary extraction methods, functional characteristics and applications of MEVs alongside examples of recent MEV modifications. Ultimately, this work aims to bridge existing knowledge gaps and facilitate the development of MEV-based therapeutic strategies.

## INTRODUCTION

Extracellular vesicles (EVs) represent a class of cell-derived nanoparticles enclosed by a phospholipid bilayer membrane. Ranging in size typically from 20 to 400 nanometers, EVs are released into the extracellular environment via specific cellular secretion pathways. These vesicles carry a complex cargo comprising nucleic acids, proteins, and lipids, which enables them to play a pivotal role in intercellular communication, biomolecule delivery and the horizontal transfer of genetic material^[1]^. Research on microbial extracellular vesicles (MEVs) has attracted growing interest in recent years, owing to their low production costs, high yield and ease of acquisition^[2]^. Different types of EVs have unique structural and functional characteristics, playing a key role in bacterial survival, environmental adaptation, and interactions between organisms. Nevertheless, the study and application of MEVs face significant hurdles. A primary challenge is the lack of standardized methods and protocols for EV isolation and characterisation, which are essential for ensuring research reproducibility and data validity. Furthermore, the specific functions and mechanisms of EVs derived from diverse microbial sources remain poorly characterized, necessitating deeper investigation into their biogenesis, biological activities, and potential clinical utility. As functional and mechanistic understanding advances, MEVs are increasingly recognized for their substantial translational potential in areas such as targeted drug delivery, gene therapy, and the discovery of novel disease biomarkers^[3,4]^. Compared with EVs derived from mammalian and plant sources, MEVs offer distinct advantages for research in fields such as gene therapy and immune modulation. Consequently, the development and optimization of efficient EV isolation techniques are paramount. This review summarizes established methods for isolating bacterial EVs, examines their biomedical applications, and outlines future research directions.

## BIOGENESIS, COMPOSITION, AND ADVANTAGES OF EVS

### Biogenesis and composition

The biogenesis of EVs is a natural biological process involving multiple formation mechanisms. Gram-negative bacteria primarily generate EVs through two pathways: one is the blebbing of the outer membrane to form outer membrane vesicles (OMVs), and the other involves the release of outer-inner membrane vesicles (OIMVs) and explosive outer membrane vesicles (EOMVs) via cell lysis^[2]^. Such mechanisms offer crucial insights into EV-mediated bacterial communication. Owing to the structural, compositional, and biogenetic differences among EVs from various species, characterizing their biogenesis pathways remains challenging^[5]^. The International Society for Extracellular Vesicles (ISEV) introduced the term “extracellular vesicles” in 2011 to describe various membrane-enclosed particles released by cells, which was later adopted in the Minimal Information for Studies of Extracellular Vesicles (MISEV) guidelines. This terminology allows for the distinction of EV subgroups based on cellular origin, size, density, surface antigens, and other characteristics^[6-8]^. Accordingly, this review uniformly categorizes bacterial EVs into OMVs, OIMVs and EOMVs (produced by Gram-negative bacteria) alongside CMVs (generated by Gram-positive bacteria).

An analysis of microbial vesicle composition reveals that their molecular cargo can be broadly categorized into three major classes: proteins, lipids and nucleic acids. Collectively, this complex array of bioactive molecules confers diverse biological functions upon the vesicles. These functions are greatly influenced by the EV composition and properties, both exhibiting significant source-dependent variability.

### Advantages of EVs

In recent years, although research on animal and plant exosomes has advanced, yet it has been accompanied by challenges such as insufficient yield, overly complex extraction methods, and ethical concerns regarding mammalian exosomes. MEVs can effectively address these problems. These vesicles have been applied in multiple fields, including drug delivery, vaccine development, disease diagnosis, and other therapeutic applications. However, despite their potential, research on MEVs remains relatively limited. Compared with exosomes from animal or plant sources, microbial-derived extracellular vesicles offer multiple advantages: easy preparation and modification, stability, and high efficiency. Additionally, ethical issues associated with using animal cell-derived exosomes can be avoided. 

## GRAM-NEGATIVE BACTERIA AND GRAM-POSITIVE BACTERIA

EVs produced by bacteria and yeast exhibit distinct differences in their generation mechanisms, compositions, and functions. Gram-negative and Gram-positive bacteria produce EVs through pathways such as outer membrane vesiculation and cell wall pore formation, while yeast forms EVs predominantly via fusion of multivesicular bodies with the cell membrane or through cell membrane budding. A recent study highlighted a novel MTT-based assay that enables rapid, reliable, and high-throughput measurement of chronological lifespan in yeast. This assay demonstrates a level of rigor and reliability comparable to traditional colony counting methods for detecting lifespan extension by caloric restriction and can effectively boost EVs production in yeast cells^[9]^. 

Small extracellular vesicles (sEVs), specifically OMVs from Gram-negative bacteria, were first identified in the 1960s using electron microscopy. They have since been detected across various conditions: in planktonic and biofilm cultures *in vitro*; in natural environments such as sewage, soil, and household dust, and within host tissues and biological fluids including cerebrospinal fluid, blood from severe infections, and gastric biopsies from Helicobacter pylori-infected patients^[10]^.

Although EVs production in Gram-negative bacteria has been observed via electron microscopy for over five decades, vesicle formation in Gram-positive bacteria was only recently demonstrated through transmission electron microscopy and proteomics^[10]^.

## EXTRACTION OF EVS

As research into EVs deepens, their potential applications are increasingly being explored. Currently, the most widely utilised EV isolation methods include ultracentrifugation, density gradient ultracentrifugation, immunomagnetic bead technology, and size exclusion chromatography. 

### Ultracentrifugation

**Figure 1:**
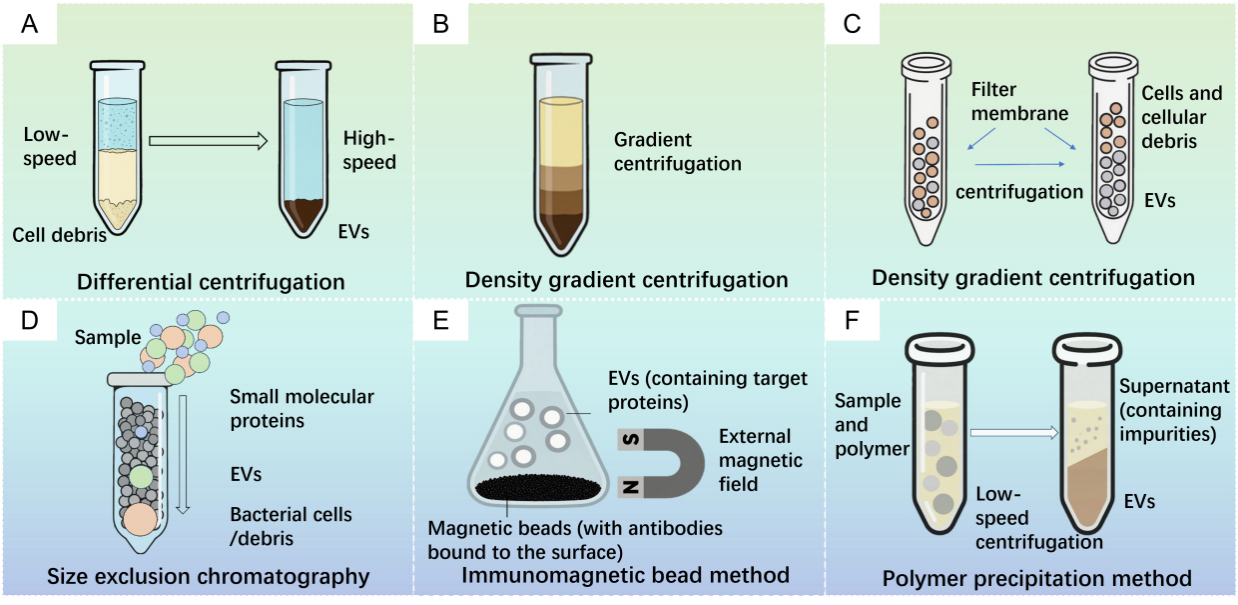
Isolation for extracellular vesicles (EVs). (A) Differential centrifugation; (B) Density gradient centrifugation; (C) Ultrafiltration centrifugation; (D) Size exclusion chromatography; (E) Immunomagnetic bead method; (F) Polymer precipitation method.

When a heterogeneous mixture (or suspension) is subjected to centrifugal force, particulate components settle according to their density, size and shape [Figure 1A]^[11]^. EVs can be separated via ultracentrifugation at speeds ranging from 100,000 ×*g* to 200,000 ×*g*^[12]^. This technique employs an optimized centrifugation process capable of generating ultrahigh centrifugal forces up to 1,000,000 ×*g* [Figure 1A]^[13]^. Despite its prevalence, this method faces limitations including prolonged processing times, susceptibility to contamination by protein aggregates and ribonucleoprotein particles, large sample volume requirements, and reduced efficiency for highly viscous or impurity-laden samples.

### Density gradient centrifugation

Density gradient centrifugation separates components with different sedimentation coefficients through centrifugation at specific speeds [Figure 1B]^[14,15]^. This method effectively isolates components by buoyant density whereby protein aggregates concentrate at the tube bottom whereas EVs band within the intermediate density region of 1.10-1.18 g/mL^[16]^. EVs isolated via gradient ultracentrifugation typically exhibit higher purity than those obtained by differential ultracentrifugation. Zhang and colleagues successfully purified PDVs using differential ultracentrifugation coupled with density gradient centrifugation, achieving high-purity preparations^[17]^. Nevertheless, this approach remains constrained by ultracentrifugation requirements and is unsuitable for large-scale production.

### Ultrafiltration

Ultrafiltration utilizes the microporous properties of specific membranes to separate vesicles from other components [Figure 1C]. This technique can be integrated with ultracentrifugation for size-based or molecular-weight-based EV separation. Similar to conventional filtration, ultrafiltration employs nanomembranes with specific molecular weight cut-offs (MWCO) to isolate EVs from complex samples^[18]^. After the removal of bacterial cells and debris, the supernatant is filtered through membranes with typical MWCO ranges of 50-100 kDa^[19]^. However, membranes with narrow pore sizes are prone to clogging and fouling by retained solutes. Additionally, hydraulic pressure during ultrafiltration may compromise the structural integrity of the vesicles^[20-22]^.

### Size exclusion chromatography

Size exclusion chromatography (SEC) separates molecular mixtures based on hydrodynamic size [Figure 1D]. Consequently, extracellular components such as vesicles, apoptotic bodies and protein aggregates gradually migrate to layers corresponding to their buoyant densities^[23]^. However, the EVs suspended in the liquid matrix may become compressed and deformed while passing through the packing material, potentially compromising their integrity. Owing to its simple operation, relatively rapid processing capabilities, and mild separation conditions, SEC has become an important tool for biomolecule purification and analysis^[24]^.

### Immunoaffinity separation

Immunoaffinity separation employs submicron magnetic beads coated with antibodies [Figure 1E], which can specifically recognize surface proteins of EVs. Following the incubation of these magnetic beads with the sample, magnetic bead-EV complexes are formed, and then EVs are separated via the application of a magnetic field^[25,26]^. Alves and colleagues utilised affinity separation technology to extract OMVs containing OmpA-His6 from the culture medium of *Escherichia coli *(*E. coli*). This indicates that affinity separation technology is also suitable for extracting MEVs with His tags^[27]^. This method can specifically separate EVs and their subtypes by recognizing and binding to surface proteins of EVs. Furthermore, the external magnetic field is readily adjustable, offering high capture efficiency, remarkable sensitivity and excellent enrichment capabilities^[28,29]^.

### Precipitation method

Precipitation techniques exploit solubility differences for biomolecule isolation [Figure 1F]^[30]^. Wei *et al.* demonstrated that ε-poly-L-lysine (ε-PL) effectively precipitates EVs at low centrifugal forces (10,000 ×*g*), offering both high efficiency and cost-effectiveness^[26]^. Separately, Shin *et al.* established a polyethylene glycol and dextran aqueous two-phase system for the separation of EVs from proteins, achieving an EV recovery of approximately 70% in the dextran phase at optimized polymer concentrations^[31]^. However, elevated salt concentrations in this method complicate downstream processing.

### Other methods

Recent advancements in separation technologies have driven the continuous emergence of novel EV separation methods. For example, field flow fractionation separates EVs based on size and molecular weight by applying a perpendicular force field. Asymmetric flow field-flow fractionation (AF4) has recently been applied in EV separation. Microfluidic technology uses microchannels to achieve separation through immunoaffinity or physical fields^[32,33]^. This approach offers distinct advantages including reduced sample volume requirements, low operational costs, high throughput capabilities and remarkable accuracy. However, this method also has limitations. For instance, highly viscous biological fluids may clog the microchannels^[29]^. Therefore, selecting an appropriate separation method is essential to obtain high-yield, high-purity, and functionally intact MEVs.

In this study, the advantages and disadvantages of the aforementioned methods are summarized in the following table [Table 1].

**Table 1:** Advantages and disadvantages of BEV separation methods

**Extraction methods**	**Advantages**	**Disadvantages**	**Ref.**
Differential centrifugation	Simple operation; allows large-volume processing; minimizes cross-contamination	Low purity and specificity; requires an ultracentrifuge; repeated centrifugation may damage vesicle structure; time-consuming (> 4 h)	[13,33,34]
Density Gradient Centrifugation	Simple operation, avoid cross-contamination, high purity	Time-consuming, high instrument cost, may cause structural damage and lipoprotein co-separation, more complex operation steps, needs ultracentrifuge	[35,36]
Ultrafiltration	Low pressure operation, good purification effect,low equipment cost, good portability, fast and easy preparation, both small and large sample volume	May alter vesicle structure; low specificity; potential sample loss due to membrane clogging	[37,38]
Polymer precipitation	Easy operation, high efficiency, using ordinary equipment, suitable for both small and large sample volume	Low purity, residual polymer (e.g., PEG) increases viscosity and requires extensive cleanup; low specificity; time-consuming (> 12 h)	[11,38,39]
Size-Exclusion Chromatography	Fast, simple, low cost, ensure the integrity and uniformity of exosome structure, high purity, Capable of processing all type of samples, both small and large sample volume	Moderate to high device cost; low specificity	[40,41]
Immune magnetic bead technology	Easy adjustment, good enrichment effect, high-purity, easy to use, no chemical contamination	High cost; low throughput and yield; elution steps may compromise vesicle structure and bioactivity	[42,43]
Microfluidics-Based Techniques (Emerging)	Cost efficient, cost-effective, portable and integrable, fast preparation, high purity	Limited sample capacity; often requires specialized devices; not yet universally applicable; fabrication can be complex	[23,34]

BEV: Bacterial extracellular vesicle.

## THE INFLUENCE AND ROLE OF SYNTHETIC BIOLOGY TECHNOLOGIES OR PLATFORMS ON EVS

Extracellular vesicles research has advanced rapidly, driven by continuous progress in separation and characterization techniques. Optimization of methods such as ultracentrifugation, density gradient centrifugation, and size exclusion chromatography has enhanced the efficiency and purity of EVs separation^[44,45]^. At the same time, the application of techniques like nanoparticle tracking analysis, flow cytometry, and electron microscopy has made precise characterization of EVs feasible^[46]^. These technological developments have established the foundation for using EVs in disease diagnosis, for example, the use of EV miRNAs as early diagnostic markers for cancer^[47]^. In therapy, EVs serve as natural nanocarriers with advantages such as favorable biocompatibility and potent targeting capabilities, which have rendered them a focal point in drug delivery system research^[48]^. Nevertheless, EVs research and application still encounter numerous challenges, including low yield, high heterogeneity, inadequate targeting, and poor functionality. These issues constrain the large scale application of EVs in clinical and industrial settings.

**Figure 2:**
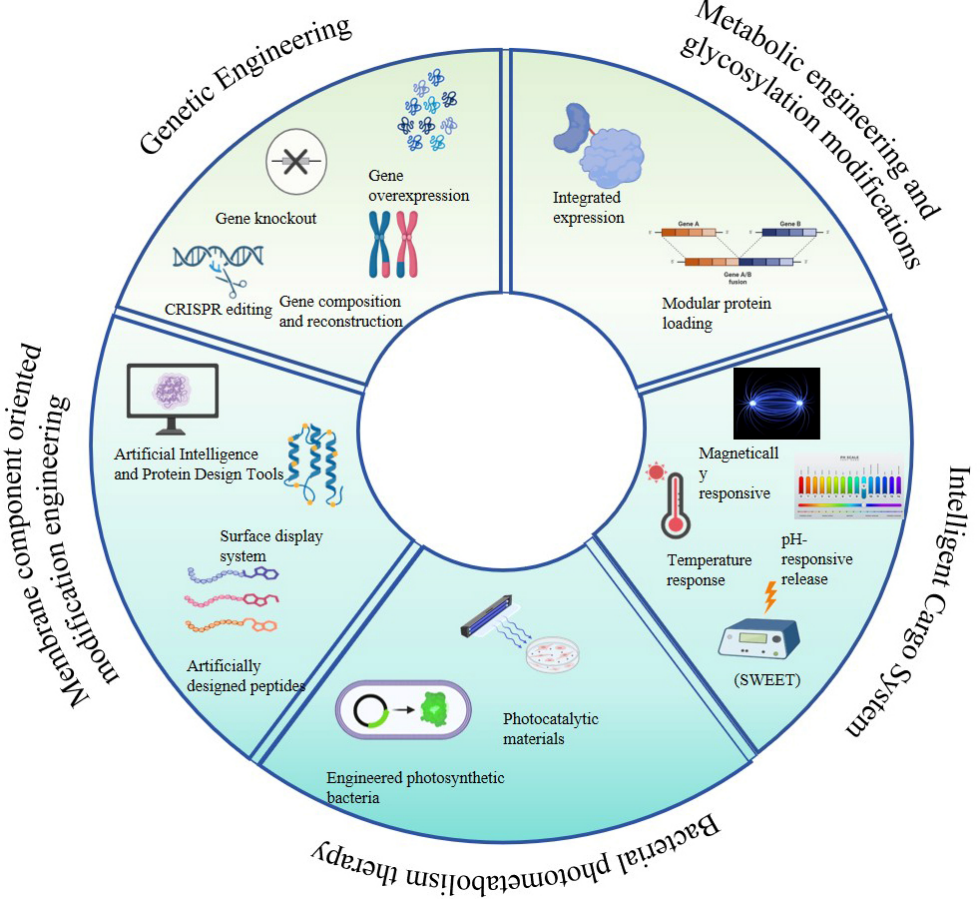
The impact of synthetic biology technologies or platforms on EVs. EV: Extracellular vesicle.

Synthetic biology is an emerging interdisciplinary field that aims to design and construct biological systems with new functions^[49]^ [Figure 2]. Its core concept involves applying engineering principles to biological research, enabling the creation or modification of biological systems through standardization, modularization, and rational design. Technologies such as CRISPR/Cas9 gene editing, metabolic engineering, and synthetic gene circuits provide powerful tools for engineering EVs and enhancing their functionality. The application of synthetic biology technology in EVs research holds broad prospects. Firstly, through cell engineering and culture condition optimization, the yield and quality of EVs can be significantly improved. Secondly, gene editing technology can be utilized to achieve precise modification of EVs surface proteins, enhancing their targeting ability and functionality. In addition, synthetic biology methods can optimise the cargo of EVs, for instance, by designing specific RNAs or proteins for enrichment within EVs^[31,50]^. The application of these technologies not only helps to enhance the value of EVs in basic research but also paves the way for the large scale application of EVs in clinical treatment and biotechnology.

### Modification of chassis cells

Synthetic biology has greatly expanded the number of gene regulatory elements (such as promoters) and other functional genetic elements (such as periplasmic localization tags)^[51,52]^. Their modular (reusable) nature, combined with compatibility with high-throughput DNA assembly methods (e.g., Golden Gate)^[53,54]^, has opened up nearly unlimited possibilities for modifying OMV-producing strains.

#### Genomic multi-target editing technology

Gene editing tools such as ZFN, TALEN and CRISPR-Cas9 have broadened targeted genome modification across many species and genes. However, ZFNs and TALENs are relatively complex and costly to construct. CRISPR-Cas9 is easier to use but off-target effects remain a concern. Combining gene editing with EV modification enables various modes of editing including single base substitution^[55]^, insertion or deletion of specific base sequences^[56]^, translational editing^[57]^, and combined inactivation or activation of multiple targets^[58,59]^ can be achieved. This combination can not only regulate the cargo and surface-modifying molecules of vesicles but also modify the overall metabolic performance of cells, ultimately enhancing the production of high-quality extracellular vesicles. Nevertheless, this field is still at an early stage with challenges, such as how to increase the yield and purity of EVs how to ensure the safety and effectiveness of gene editing tools, and how to achieve precise delivery and regulation *in vivo*.

However, the development prospects in this field remain very broad. Zanella and colleagues constructed a *BL21(DE3)Δ60* engineered strain through CRISPR/Cas9-based genome editing, which improved both recombinant protein expression and OMVs loading efficiency^[60]^. In archaea, CRISPR-Cas9 gene editing of methanogenic archaea was reported for the first time in 2023, and targeted knockout of *Msv_1527* genes resulted in a 100% increase in yield of EVs^[61]^. In fungal systems, a specific CRISPR-Cas9 editing platform inhibited *PMA1 *gene expression in *Candida albicans* and significantly reduced *PMA1* protein loading in EVs, providing a new technical path for targeted therapy of inflammatory bowel disease^[62]^. These research results not only establish a precise regulation system for the production and function of EVs, but also open new research directions for microbial engineering in biomedicine.

#### Genome synthesis and reconstruction

Breakthrough advances in synthetic biology have significantly enhanced humanity’s ability to rationally design and selectively modify life systems. In microbial genomic engineering, research teams have successively achieved whole genome synthesis technologies for both prokaryotes and eukaryotes. They have verified the biological functional integrity of synthetic life systems through artificial genome reconstruction. Notably, artificial chromosome reconstruction technology has shown strong genetic reshaping potential, with its dynamic recombination characteristics providing a new regulatory dimension for the directed evolution of cellular metabolic networks^[63,64]^. Relying on the genomic synthesis technology platform, researchers can systematically reshape the biosynthetic pathways of EVs by optimizing the protein expression regulatory network and constructing a framework for the sequential expression of non-coding RNAs, thereby establishing an intelligent vesicle generation system. This innovative approach, which integrates synthetic biology with systems biology, will effectively shorten the development cycle of engineered strains with specific industrial characteristics.

For the *E. coli *Nissle 1917 (EcN) chassis system, researchers have used genome streamlining engineering to delete non-essential gene clusters. This approach has significantly improved the biosynthesis efficiency of OMVs^[65]^. Fusion antigen of influenza virus, human papillomavirus, pneumococcus, *Staphylococcus aureus*, and *Acinetobacter baumannii* displayed on OMVs produced by *E. coli *through genetic engineering can stimulate the body to produce specific antibodies against these pathogenic microorganisms, thereby playing an effective preventive role^[66,67]^. These results fully demonstrate the multifunctional application value of genome editing technology in the engineering transformation of MEVs, which not only optimizes the yield of EVs, but also provides important technical support in terms of immunogenicity enhancement and regulation of physical properties.

Beyond direct genomic modifications of engineered bacteria, various engineered bacteria can now be designed and constructed. This enables the synthesis and release of vesicles *in vivo*, facilitating biomedical applications such as disease prevention and proactive health interventions. Zhou* et al.* developed an orally administrable engineered probiotic that overexpresses catalase and superoxide dismutase to eliminate reactive oxygen species, thereby improving the treatment of intestinal inflammation^[68]^. The “protective suit” system for live bacteria established by Liu shows promise in providing new strategies for bacterial transplantation and prevention of enteritis^[69]^. Cui *et al.* have been conducting long-term research on optogenetic engineered intestinal bacteria as living drugs^[70]^. By empowering engineered bacteria with synthetic biology techniques, they have expanded the applications of these bacteria in areas such as enteritis, gut-brain axis regulation, gut-kidney axis regulation, and blood glucose control^[71-73]^. The customization, efficacy enhancement, and safety control modifications of engineered bacteria and their EVs will provide robust support for the biomedical applications of *in situ* synthesized EVs *in vivo*.

This study summarizes some strategies and effects of using genetic engineering methods to modify chassis cell genes [Table 2].

**Table 2:** Application of genetic engineering methods in extracellular vesicles

**Method**	**Technology**	**Function**	**Effect**	**References**
Genetic Engineering	CRISPR editing	Knockout 59 endogenous OMV-cargo protein genes in *BL21(DE3)Δ60 Escherichia coli*	Increase the level of recombinant protein	[60]
		Knockout of the Msv_1527 gene in methanogenic archaea	EV production doubles	[74]
		PMA1 expression in Candida albicans was knocked out using a Crispr-Cas9-based fungal strain editing system	For colitis treatment	[62]
	Gene composition and reconstruction	Genome streamlining engineering directed deletion of non-essential gene clusters	Improve the biosynthesis efficiency of extracellular membrane vesicles (OMVs)	[65]
		Combination of different strains’ OMVs	Expand vaccine coverage	[66]
	Gene knockout	Knockout of the Fks1p and Chs3p genes in *yeast* cells	Increase in OMVs production	[75]
		Introducing the curvature regulation mechanism of eukaryotic cell membranes into *Escherichia coli*	BMV production increased by 149 times	[76]
		Knockout lpxP in the plague bacterium KIM6 and insert lpxE	Increase in OMVs production	[77]
		Knockout the ΔtolB gene	Increased vesicle formation rate	[78,79]
		Inactivated coding genes of lipid A acyltransferases, such as msbA, msbB, lpxL1, and lpxM	Reduce LPS toxicity	[80-82]
	Gene overexpression	Overexpression of catalase and superoxide dismutase	Improve intestinal inflammation	[68]
		Using genetically modified bacterial strains expressing tyrosinase to produce encapsulated biological polymer melanin (OMV^Mel^) in OMVs	Achieve non-invasive monitoring of the distribution of OMVMel related to tumors in the body	[83]
		Engineering the overexpression of the LcrV antigen from Yersinia pestis in pCD1-deleted plague bacteria	Significantly enhance the immunogenicity of BMVs	[77]

OMV: Outer membrane vesicle; BMV: Bacterial membrane vesicle.

### Targeted modification engineering of membrane components

Targeted modification of membrane components is one of the key strategies for optimizing the function of OMVs. By engineering membrane-associated proteins, lipids, or other surface molecules, the biological properties of OMVs can be precisely regulated. These properties include targeting ability, stability, immunogenicity and drug loading efficiency. 

#### Artificial design of vesicle surface proteins

In recent years, the rapid development of computational biology and AI in protein science has provided powerful tools for the rational design of membrane proteins. Advanced protein structure/function prediction tools such as AlphaFold^[84]^, protein large language models^[85]^, and others (e.g., Rosetta, RFdiffusion^[86]^) have enabled researchers to more accurately predict and design novel membrane proteins, even achieving complete de novo design.

These techniques have been successfully applied to several OMV related research cases, including the application of AlphaDesign to design *in vivo* active inhibitors of the phage defense protein RcaT-Sen2^[87]^. In membrane fusion mechanism innovation, the Severe Acute Respiratory Syndrome Coronavirus 2 (SARS-CoV-2) like spike proteintransmembrane domain designed based on the evolutionary scale modeling 2 (ESM 2) language model enables OMV to achieve efficient membrane fusion with angiotensin-converting enzyme 2 (ACE2) expressing cells^[88]^. Functionalized OMVs have also shown great potential in cancer diagnosis and treatment. The antimicrobial peptide Polybia-mastoparan I (MPI) loaded in* E. coli* OMV provides a novel delivery vehicle for bladder cancer treatment^[89]^; The fusion expression of pH-sensitive green fluorescent protein and EVs membrane proteins such as CD63 enables EVs to achieve fluorescence signal activation in the acidic tumor microenvironment, providing a real time visualization tool for cancer diagnosis and treatment monitoring^[90]^. In the future, with the development of multimodal AI protein design tools, artificial design of vesicle surface proteins will achieve higher precision functional programming, promoting the application of OMVs in precision medicine. These breakthrough advances not only demonstrate the synergistic effects of computational biology, protein engineering, and nanotechnology but also lay a solid technical foundation for the intelligent design and clinical translation of OMVs.

#### Analysis and prediction of protein structure and specific binding design

In protein structure prediction, elucidation and specific molecular design, several recent studies have promoted breakthroughs in the biomedical application of OMVs through the integration of computational biology, synthetic biology, and nanobiotechnology. Researcher used synthetic biology technology to construct recombinant probiotics and modify membrane components to display BMP-2 and CXCR4 on the surface of bacterial extracellular vesicles (BEVs), achieving dual functions of bone targeting and bone regeneration, thereby providing an innovative treatment for osteoporosis^[91]^. Other researchers engineered EVs by fusing HaloTag with vesicle anchoring proteins on the EV surface, enabling the targeted display of molecules such as GalNAc to successfully target human liver cells^[92]^.

Moreover, the integration of optogenetics technology and bioelectronic medicine has opened up new paths for disease diagnosis and treatment. Optical imaging detection and bioelectronic drugs constructed using light-controlled engineered bacteria have enabled precise monitoring and dynamic intervention for chronic diseases including kidney disease^[72]^. In tumor immunotherapy, two innovative strategies demonstrate the unique advantages of OMVs as nanoscale immune adjuvants. Firstly, OMV surface nanobodies serve as biological scaffolds that activate immune cells through specific binding to tumor-associated antigens, providing a targeted immune activation strategy for solid tumor treatment^[93]^. Secondly, engineered photosynthetic bacteria and their OMVs are designed as intelligent antigen capture systems, capable of efficiently enriching tumor antigens and delivering them to the tumor-draining lymph node (TDLN) region, thereby enhancing the body’s anti-tumor immune response^[94]^. Together, these studies form a complete technological chain - from protein structure prediction to functionalized vector design, and then to precise disease intervention. This work deepens our understanding of the biophysical properties of OMVs and provides theoretical support and technical paradigms for developing next-generation intelligent biologics.

### Cell processes regulated by molecular encapsulation

Organoid extracellular vesicles (OEVs) as natural nanocarriers, have demonstrated immense potential in the field of drug delivery. The introduction of synthetic biology technology has revolutionized the cargo-carrying capacity of OEVs, achieving significant breakthroughs, particularly in the encapsulation of long-sequence nucleic acids and functional metabolites. Utilizing synthetic biology strategies, OEVs have made progress in encapsulating macromolecular nucleic acids and hydrophobic metabolites.

#### Engineering loading of long-sequence nucleic acids and RNA-binding proteins

Using synthetic biology methods, researchers have achieved multilevel optimization design. Precise regulation of the spatial arrangement of 5'UTR, 3'UTR and IRES elements has increased target protein expression levels by thousands of times. Polycistronic mRNA technology enables a single mRNA molecule to encode multiple functional proteins, such as components of the CRISPR-Cas9 system. Specific RNA secondary structures can enhance miRNA binding capacity, achieving efficient gene regulation. These strategies provide important technical support for the functional modification of EVs.

Cui *et al.* proposed an economical and efficient technology to prepare engineered OMVs by overexpressing pre-miRNA in *E. coli*^[95]^. Similarly, Santos *et al.* demonstrated that exogenous loading of miR-195-5p, a tumor suppressor miRNA, into EVs enhances its anti-tumor activity and improves targeted therapy response in melanoma against patients with *B-raf* proto-oncogene mutations^[96]^. This finding highlights the potential of EV based strategies to enhance clinical outcomes. These studies indicate that through genetic modification and physical loading methods, OMVs can be designed as efficient anticancer carriers, providing new ideas and technical means for developing novel cancer treatments. However, issues such as delivery efficiency, functional stability, immunogenicity, and production costs still need to be addressed further to realize their clinical translation potential.

Mammalian cells contain over 500 types of RNA binding proteins (RBPs), providing a rich source of natural regulatory elements for synthetic biology^[97]^. Bioengineered, enhanced EVs can deliver collagen mRNA into the skin, promoting the production of collagen lost due to aging in recipient skin cells^[98]^. For surface functionalization, the fusion system of the bacterial membrane protein ClyA and SnoopCatcher enables the modular capture of vesicle surface proteins, and this strategy, combined with intestinal *in situ* synthesis technology, can dynamically release engineered EVs in the tumor microenvironment to enhance antitumor activity^[99,100]^. Zhuang and colleagues further crossed species boundaries by constructing photodynamic hybrid vesicles (BPNs) that fuse plant thylakoid membranes with bacterial OMVs. These vesicles generate reactive oxygen species to directly ablate tumor tissues under laser irradiation^[101]^. In addition, a synergistic strategy of chemical modification and bioengineering showed unique advantages: Shen *et al.* integrated the tyrosine rich protein statherin into the surface of OMVs, and significantly extended its blood circulation time and increased tumor accumulation rate through polyethylene glycol modification^[102]^. These multidimensional engineering strategies reveal the molecular design potential of EVs as “programmable biological carriers”. They also provide an innovative technical paradigm for the integration of drug delivery, immune regulation, and real-time diagnosis and treatment in precision medicine.

#### Targeted loading of functional metabolites

The design of membrane proteins for metabolite loading requires comprehensive consideration of multiple key factors, including balancing hydrophobicity and membrane stability, maintaining metabolite binding activity while ensuring correct folding, and introducing environmentally responsive conformational change modules. Synthetic biology strategies have shown great potential in achieving efficient metabolite enrichment. Specific approaches include construction of membrane localised metabolic factories, development of metabolite capture and fixation systems, and establishment of dynamic regulatory networks such as the use of quorum sensing to control the timing of metabolite synthesis.

In targeted modification, various innovative strategies have been successfully applied to the functional customization of engineered OMVs. Wang *et al.* developed an OMV-cancer cell membrane hybrid carrier coated on hollow polydopamine (HPDA), which was also used for tumor imaging^[103]^. Rezaei *et al.*^[104]^ and Sepahdar *et al.*^[105]^ introduced OMVs expressing ClyA-EGFR scFv, which demonstrated high affinity for EGFR-positive cancer cells both *in vitro* and *in vivo*. Additionally, OMVs overexpressing ClyA-Hy can target hypoxic tumors and remodel the tumor matrix^[106,107]^.

Besides ClyA fusion proteins, modifying OMVs with glycosylphosphatidylinositol (GPI) anchored proteins is another promising approach. Marianne *et al.* reported that the lipid portion of GPI anchors inserts into the cell membrane, and two completely different GPI proteins can be displayed on the same surface of OMVs^[108,109]^. These multidimensional engineering strategies enhance both metabolite loading efficiency and targeted delivery capabilities of OMVs. They also provide new technological platforms for tumor imaging, treatment, and microenvironment regulation. These advancements demonstrate the broad application potential of synthetic biology in precision medicine, paving new paths for future biomedical research and clinical applications.

#### Intelligent cargo loading system

In the field of intelligent drug delivery system development, the deep integration of synthetic biology and membrane vesicle engineering is promoting the innovation of drug delivery technology in the direction of precision and controllability. Researchers have built a synthetic OMV cargo platform that is completely decoupled from the host biochemical network, and its drug delivery module can be fully manually designed.

Chen and colleagues designed an OMV based multi module system that uses charge reversal polymers to separate functions, targeting both malignant and immune cells^[110]^. In another approach, EVs spontaneously hybridise with nonlamellar liquid crystalline lipid nanoparticles (LCNPs) to form hybrid extracellular vesicles (HEVs). This allows efficient loading of siRNA, mRNA, and nucleic acid aptamers under mild conditions^[110]^. In addition to strategies that exploit the physicochemical or biological properties of specific tissues or cells, targeted delivery can also be achieved with the help of external forces, such as the system formed by doxorubicin loaded into placental mesenchymal stem cell derived exosomes and modified with carboxylated Fe_3_O_4_ nanoparticles, creating an Exo-Dox NP system^[111]^. In terms of cargo efficiency improvement technology, a breakthrough has been made in the development of the “Shock Wave Extracellular Vesicle Engineering Technology” (SWEET), which uses shock waves (SWs) to achieve efficient encapsulation of siRNAs^[112]^. The bacterial biomineralization system uses a mineral crystal deposition strategy to achieve drug-loading and bactericidal synergy while scavenging *Staphylococcus aureus*^[113]^.

These innovative technologies rely on the multidimensional collaboration of modular biological components, including biosensors, molecular switches and orthogonal synthesis systems. They also integrate physical cargo delivery strategies such as electroporation and microfluidics. Together, they redefine OMVs as intelligent nanorobots and lay the technical foundation for the fully artificial construction of synthetic MVs. This enables programmable control from gene circuit design to cargo vesicle assembly, promoting the integration of precision medicine towards molecular level diagnosis and treatment.

### Improvement of EV production and quality

#### Increase production

In large scale OMV production, the collaborative innovation of strain metabolic engineering and membrane biophysical regulation is driving the simultaneous increase in yield and quality. Researchers have reconstructed bacterial vesicle generation through multidimensional strategies. A team from the University of Waterloo and the Southern University of Science and Technology introduced the eukaryotic membrane curvature regulator EutS into *E. coli*. This protein induces local outer membrane bending, increasing OMV yield 149-fold while reducing vesicle heterogeneity through optimised membrane tension balance^[76]^. At the lipid metabolism level, Wang *et al.* performed precise gene editing on *Yersinia pestis* KIM6^+^. They knocked out the lipid A acyltransferase gene lpxP and inserted the phosphatase gene lpxE. This increased OMV yield 2.3-fold and improved biosafety by removing the toxic phosphate group from lipid A^[77]^. 

These advances reveal three core mechanisms for yield enhancement. First, remodeling membrane structural dynamics using curvature regulating proteins such as EutS and the MVSP family. Second, optimising outer membrane stability and vesicle budding efficiency by targeting the lipopolysaccharide synthesis pathway, for example through mutations in waaP or lpxL. Third, lowering the vesicle release energy barrier by adjusting cell wall mechanical strength, such as reducing peptidoglycan crosslinking via dacB knockout. When combined with immunogenicity optimisation strategies, including surface display of meningococcal NadA antigen or chimeric TLR4/MD2 agonist peptides, engineered OMVs can achieve the triple goals of high yield, low toxicity and potent immune activation.

In the future, machine learning assisted design of membrane protein allosteric elements, intelligently coupled with quorum sensing systems such as LuxR and LuxI, may pioneer a third generation OMV biomanufacturing paradigm. This would enable on demand production and directed release.

#### Optimize cell culture conditions, such as designing specific medium components or bioreactor systems

In large scale production of engineered EVs, the integration of culture condition optimization and advanced bioreactor technology is driving the innovation of production processes towards precision and controllability. By systematically regulating bioreactor parameters, such as increasing the dissolved oxygen content from 30% to 150% in the *Neisseria meningitidis* culture system, the bacterial oxidative stress pathway can be activated, leading to a fourfold increase in OMVs production^[114]^. This metabolic stress based strategy can be combined with optimisation of the carbon to nitrogen ratio, for example by reducing the C/N ratio to induce membrane remodeling, and with regulation of quorum sensing molecule gradients such as 3OC_12_-HSL. Together these form a multidimensional yield enhancement scheme.

Furthermore, the introduction of optogenetic regulation systems has created a new dimension of spatiotemporal precision control. Cui *et al.* ^[70]^developed conversion microgels (UCMs) that activate programmed colonisation of engineered light responsive bacteria (Lresb) in the intestine through a near infrared to blue light conversion system. The cell adhesion agents secreted by these bacteria increase intestinal colonisation efficiency of EcN 3.2 fold, significantly alleviating DSS induced colitis in mice. This technology platform has been further extended to the field of tumor treatment, achieving a triple synergistic effect of photothermal ablation, gene silencing andimmune activation by simultaneously secreting immune stimulators (such as IL-12) and RNA-loaded OMVs through light controlled engineered bacteria^[115]^. 

These innovative strategies break the limitations of traditional static culture. They construct an intelligent biomanufacturing system with awareness, response, and output capabilities through dynamic regulation of dissolved oxygen, light signals, and quorum sensing molecules. This provides a programmable solution for industrial production and precision medicine applications of EVs. In the future, combining machine learning algorithms to optimise bioreactor parameters in real time will shift EV manufacturing from experience driven to data driven paradigms.

## Design intelligent biomaterials to achieve automatic separation and enrichment of EVs

In the field of EVs engineering technology driven by intelligent biomaterials, innovative separation, detection, and functionalization strategies are propelling rapid progress towards precision medicine applications. Li *et al.*^[116]^ has developed zwitterionic coordination separation technology. By simulating the charge distribution characteristics of phosphatidylcholine (PC) on the cell membrane surface, this technology utilizes zwitterionic ligands to form a high-affinity dynamic bonding network with EVs membranes, achieving single-step rapid separation of EVs in biological fluids. This lays the foundation for subsequent functional analysis^[116]^. The same team then constructed a pH responsive block copolymer assembly system. By precisely adjusting the pH between 5.0 and 8.0, they altered the hydrophilic hydrophobic balance on the EV surface. This triggered self-assembly of discrete nanoscale EVs (around 100 nm) into micron scale clusters (around 2μm), enhancing membrane protein detection sensitivity by three orders of magnitude and enabling trace biomarker analysis^[117]^.

For therapeutic applications, engineered bacterial microcapsule technology overcomes traditional drug delivery limits. By integrating a light controlled lysis gene circuit into EcN, researchers created a subcutaneous implant that programmatically regulates the production and release cycle of therapeutic proteins such as IL-10. This achieved sustained release treatment for up to 28 days in inflammatory bowel disease models^[118]^. For the diagnosis of neurodegenerative diseases, an EVs immune capture chip based on α-synuclein-specific antibodies, combined with single vesicle mass spectrometry analysis technology, can detect these EVs in the cerebrospinal fluid of Parkinson's disease patients^[119]^. These breakthrough technologies, through intelligent design of material-biological interface engineering, have constructed a full-chain solution from separation and purification through functional enhancement to clinical translation, marking a leapfrog development of EVs engineering from a laboratory tool to clinical grade diagnostic and therapeutic platforms.

This paper lists some applications of oriented modification of membrane components and metabolic engineering for bacterial outer membrane vesicles [Table 3].

**Table 3:** Application of membrane component targeted modification engineering and metabolic engineering in extracellular vesicles

**Method**	**Technology**	**Function**	**Effect**	**References**
Membrane component oriented modification engineering	Artificial Intelligence and Protein Design Tools	Transmembrane domain of spike protein similar to SARS-CoV-2 designed based on the ESM-2 language model	Make OMV efficiently fuse with cells that highly express ACE2	[88]
		Fuse and express pH-sensitive green fluorescent protein with EV membrane protein	Used for cancer diagnosis and treatment monitoring	[90]
		Load Polybia-mastoparan I (MPI) fusion peptide into *E. coli* OMVs	Used for clinical treatment of bladder cancer	[89]
	Surface display system	Displaying BMP-2 and CXCR4 on the surface of BEVs through membrane component modification	Achieve dual functions of bone targeting and bone therapy	[91]
		Fuse targeting ligands such as sugar GalNAc into EVs so that they are displayed on the EV surface	EV successfully targets human liver cells	[92]
		Photodynamic hybrid vesicles (BPNs) were constructed by fusing plant thylakoid membranes with bacterial OMVs	Under laser irradiation, reactive oxygen species can be generated to directly ablate tumor tissue	[101]
		Integration of the tyrosine-rich protein statherin into the surface of OMVs	Increase tumor accumulation rate	[102]
Metabolic engineering and glycosylation modifications	Integrated expression	OMV surface displays synthetic antigen-binding proteins (SNAP) containing streptavidin-binding domains and outer membrane scaffold proteins	Achieve efficient loading of biotinylated antigens	[120]
		Using OMVs from Salmonella typhimurium to deliver type 2a O-polysaccharide antigens of Shigella dysenteriae	Prevention of type 2a Shigella dysenteriae infection in mice	[121]
	Modular protein loading (SpyTag/SpyCatcher)	Fusion of SpyTag with Hbp does not affect the localization of Hbp in BMVs, and SpyCatchers carrying different antigens can directly connect with SpyTag on BMVs, thereby loading the target antigens onto the BMVs	Effectively stimulate the body’s immune response	[99,122]
		Fuse RGD oligopeptide with bacteria ClyA	RGD peptides target and enrich in immune cells, tumor blood vessels, and tumor cells	[123]
		The bacterial membrane protein ClyA anchors the nano capture agent SnoopCatcher to the vesicle surface. This captures the corresponding tagged fusion purified protein and enables modular vesicle surface modification	Real-time *in situ* synthesis and release of vesicles in the intestine for anti-tumor research	[99,100]

OMV: Outer membrane vesicle; SARS-CoV-2: Severe Acute Respiratory Syndrome Coronavirus 2; ACE2: angiotensin-converting enzyme 2; ESM: evolutionary scale modeling; BMV: *E. coli*: *Escherichia coli*.

## APPLICATIONS OF EXTRACELLULAR VESICLES IN MEDICINE

### Drug delivery and therapeutic platforms

**Figure 3:**
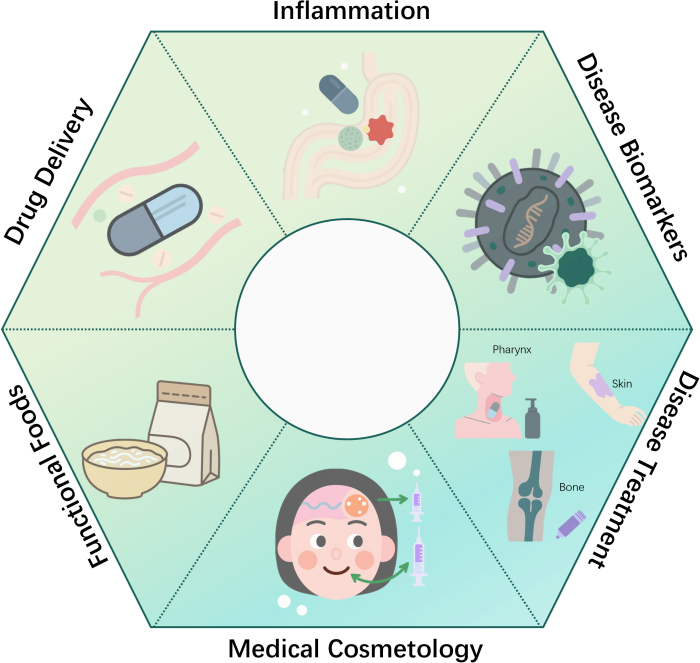
Applications of microbial extracellular vesicles in medicine.

Biomedical engineering advancements have established drug delivery systems as pivotal for enhancing therapeutic efficacy. Brezgin *et al.* (2024) highlighted that EVs have emerged as a highly promising drug delivery platform, attributed to their excellent biocompatibility, low immunogenicity, and ability to cross biological barriers^[124]^. Proteins, RNAs, and small-molecule drugs can be loaded into EVs through either endogenous or exogenous approaches. Furthermore, tissue-specific delivery of EVs can be achieved by leveraging surface display technologies, such as modification with targeting peptides, antibodies, or glycan chains. Currently, EVs are being utilized in clinical trials for the treatment of tumors, inflammatory diseases, and other conditions. However, their large-scale production, drug loading efficiency, and targeting stability remain key challenges to date [Figure 3]^[125]^. MEVs demonstrate significant potential as dual-purpose drug carriers and therapeutic platforms, leveraging their inherent multifunctionality. Engineered MEVs enable efficient delivery of diverse therapeutics such as chemotherapeutics and antibiotics, while offering novel strategies for cancer intervention. Tumors have remained a grave threat to human health in recent years, becoming the leading cause of death. Jan and colleagues found that paeoniflorin can precisely modulate key oncogenic processes via multiple pathways, positioning it as a highly promising low-toxicity, high-efficacy natural anticancer candidate^[126]^. While comparable to conventional liposomes in size and structural profile, MEVs exhibit greater bilayer complexity with diverse lipids/proteins alongside internal cargo and surface molecules. Crucially, specific surface components facilitate targeted cellular delivery^[40]^. Further advantages include passive tumor accumulation via the enhanced permeability and retention (EPR) effect for site-specific drug deposition. Additionally, these vesicles offer enhanced immune recognition through pathogen associated molecular patterns that promote uptake by neutrophils and macrophages. Furthermore, they provide intrinsic drug protection against degradation. Harnessing MEVs as delivery vehicles thus expands the therapeutic arsenal with promising clinical translation potential^[26]^.

As the understanding of the unique biological properties of MEVs deepens, researchers are exploring optimized therapeutic drug encapsulation within these natural nanocarriers. Effective drug loading strategies are critical for ensuring MEV delivery system efficacy and safety. Current methodologies primarily comprise endogenous and exogenous approaches. Endogenous loading integrates therapeutics during vesicle biogenesis, while exogenous methods introduce drugs into isolated MEVs via specific techniques. The selection between these complementary strategies depends on drug characteristics, required release kinetics and intended therapeutic outcomes.

Exogenous loading introduces therapeutics into pre-isolated EVs post-purification. While diverse physicochemical techniques such as electroporation, incubation, sonication, extrusion and freeze thaw cycles enable exogenous drug incorporation, their efficacy varies significantly^[39]^. These methods risk inducing the aggregation of EVs and drugs as well as altering vesicle properties. Conversely, endogenous loading exploits cellular sorting mechanisms to concurrently produce biomolecules within vesicles. For instance, transfected cells or cells incubated with cargo utilize endogenous pathways to package therapeutics into EVs for extracellular secretion^[39]^. Alternatively, engineering cells to stably express RNA and protein therapeutics facilitates active cargo loading through enhanced fusion with molecules enriched in EVs.

Further research regarding BEV drug loading strategies has revealed the potential of these natural nanocarriers in cancer treatment. Compared with live or attenuated bacteria, MEVs are considered safe because they cannot replicate autonomously *in vivo*. Furthermore, MEVs possess high thermal stability and carry a variety of immunogenic components associated with the membrane or cytoplasm. These inherent properties enable them to enhance drug targeting, reduce side effects, and synergize with immunotherapy to improve treatment success rates.

It has been demonstrated that EVs derived from *E. coli* without lipopolysaccharide (LPS) can target cancer cells *in vivo* and alleviate tumor burden by continuously producing C-X-C motif chemokine ligand 10 (CXCL10) and Interferon gamma (IFN-γ) and triggering an antitumor immune response^[127]^. To improve the efficacy and application scope of MEVs, Chen *et al.* integrated melanoma cell membranes, *Salmonella* vesicles, and photothermal modules to inhibit tumorigenesis^[128]^. MEVs loaded with chemotherapeutic drugs enhanced the penetration of doxorubicin and tumor cell apoptosis^[129]^, indicating that this approach may be a promising targeted delivery strategy. To improve the targeting efficiency of MEVs, corresponding molecules can be conjugated to their surfaces. For example, the coating of arginine glycine aspartic acid peptides on the OMVs of *Salmonella* or *E. coli* significantly enhances their tumor targeting ability^[130,131]^.

In summary, the flourishing field of biomedical engineering has promoted the exploration of drug delivery systems, and MEVs have become promising candidates due to their unique biological properties. MEVs offer advantages such as efficient drug delivery, targeted accumulation in tumors and specific cell targeting. Compared with synthetic nanocarriers like liposomes, MEVs have similar sizes but more complex bilayer structures, making them multifunctional carriers. Strategies for loading therapeutic drugs into MEVs, including endogenous and exogenous methods, are being explored, each presenting distinct advantages. These loading technologies are crucial for ensuring the efficacy and safety of MEVs in drug delivery systems. Recent studies have demonstrated the potential of MEVs in cancer treatment, highlighting their ability to target cancer cells, alleviate tumor burden and enhance the efficacy of chemotherapeutic drugs. Further advancements, such as the modification of surface targeting molecules, are expected to improve the targeting efficiency of MEVs and expand their clinical applications. Overall, MEVs represent a promising approach for developing effective and targeted drug delivery systems in cancer treatment.

### Inflammation and immune system regulation

Bacteria-derived EVs can regulate intestinal immune homeostasis by activating or inhibiting intestinal-related immune cells, including dendritic cells, macrophages, T cells and B cells. This helps defend against pathogen infections and prevent the onset and progression of inflammatory bowel disease. According to Maerz* et al*., the intestinal symbiont *Rhodobacter cloacae* can penetrate the mucus layer of the intestinal epithelium by producing EVs^[132]^, allowing the bacteria to deliver substances involved in intestinal immune responses, thereby preventing colitis in mice. Research concerning lactic acid bacteria confirmed that the culture solution of kefir grain lactic acid bacteria can not only reduce the levels of inflammatory cytokines in a murine model of inflammatory bowel disease but also decrease serum catalase levels^[133]^. Similarly, another study found that the phage of *Lactobacillus plantarum* can reduce inflammatory cytokines, serum peroxidase levels, transmural leukocyte infiltration, and colonic goblet cell loss in a mouse model of inflammatory bowel disease, and regulate the inflammatory response. In a mouse model of atopic dermatitis induced by *Staphylococcus aureus*, phage proteins of Lactobacillus plantarum reduced epidermal thickening and IL-4 levels, indicating that lactic acid bacteria have a protective effect against atopic dermatitis^[134]^. Additionally, vesicles from *Streptococcus thermophilus* can serve as antibacterial agents, delivering bacteriocin peptides into the bloodstream. These vesicles deliver bacteriocin peptides to pathogenic lactic acid bacteria, inhibiting their growth and disrupting their membrane integrity, which may lead to changes in complex microbial communities^[135]^. However, the production of EVs may also promote the development of certain diseases. It has been reported that EVs produced by *Staphylococcus aureus *can induce the production of proinflammatory cytokines and cell death, suggesting that EVs may play a role in the pathogenicity of *Staphylococcus aureus* to the host^[136]^.

MEVs play a pivotal role in maintaining intestinal immune homeostasis. Furthermore, their immunomodulatory properties have garnered significant attention in the vaccine research field. In vaccinology, OMVs derived from pathogenic bacteria exhibit considerable potential as an immunogenic platform. These vesicles display various pathogen-related antigens in their natural conformation, effectively activating humoral and cellular immune responses. OMVs can harbour a range of antigens including proteins, lipids (such as LPS) and polysaccharides, thereby providing a natural multivalent antigen expression system to enhance immune responses mediated by T cells and B cells. Owing to their structural similarity to the parent bacteria, OMVs can effectively mimic the natural infection process, thus triggering a robust immune response. Additionally, OMVs have been shown to effectively target and activate antigen-presenting cells, especially dendritic cells, initiating an effective adaptive immune response including cytotoxic T cells and B cells^[137]^. These immune cells can produce a variety of antibodies, including IgG, IgM, and IgA^[137]^. OMV-based vaccines have proven effective in preventing various infectious diseases. For example, modified *Neisseria meningitidis* OMV vaccines are currently undergoing extensive testing and clinical trials, showing better protective effects than traditional vaccines^[138]^. A study used OMVs as an mRNA delivery platform, genetically engineering OMVs by surface-modifying RNA-binding protein L7Ae and *Listeria monocytogenes* listeriolysin O to obtain OMV-LL^[139]^, aiming to develop a new type of mRNA tumor vaccine.

In summary, MEVs can regulate intestinal immunity and be applied in vaccinology. However, translating this approach into clinical applications still faces challenges. Currently, there are no clinical trials involving BEVs for diagnosis and treatment. Therefore, ensuring the safety, scalability, and reproducibility of OMV production is crucial for its widespread application.

### Disease biomarkers

EVs can provide valuable information for disease diagnosis and prognosis by reflecting the status and function of their cells of origin. The investigation of MEVs as disease markers constitutes a rapidly growing research field. EVs carry disease-related biomarkers, and sampling from blood or other body fluids helps in early diagnosis and monitoring of various diseases.

MEVs can be isolated from cultures or biological samples and contain bacterium-specific nucleic acids (DNA and RNA), proteins, and lipids, including bacterial plasmids, viral genomes, antigenic proteins, and lipopolysaccharides. These molecules are characteristic markers of microbial EVs, reflecting the type, activity, and pathogenicity of microorganisms, including bacterial drug resistance, viral variability, and parasitic infectivity. MEVs can serve as infection markers and quality control indicators. Studies have confirmed their presence in various biological fluids, including human blood, urine, and saliva. These vesicles carry biological information or metabolic molecules from parent bacteria, reflecting the composition of the host microbiota. They actively participate in communication between bacteria as well as between hosts and bacteria, reflecting the physiological and pathological status of the host^[140,141]^. Han *et al.* analyzed the DNA epigenetic patterns of small extracellular vesicle (sEV) biomarkers, lipopolysaccharide (LPS)-positive OMV populations, and sources of specific periodontal pathogens in saliva samples from healthy individuals, as well as patients with gingivitis and periodontitis^[142]^. Their findings indicated that genome-wide sEV methylation may serve as a valuable biomarker for periodontitis.

Additionally, Yoon *et al.* isolated MEVs from urine samples of 91 colorectal cancer (CRC) patients, performed 16S rRNA gene sequencing for microbiome analysis and characterized the differential microbiota between CRC patients and healthy controls^[143]^. The study concluded that, compared with healthy controls, urinary EVs from CRC patients harbor a unique intestinal microbiome profile. Thus, microbial signatures within urinary EVs hold potential as biomarkers for CRC diagnosis.

The advantages of MEVs as disease markers include their biological activity and diversity, the capacity to carry multiple molecules related to diseases, the potential to provide non-invasive diagnosis and the ability to reflect the complex interactions between the host and microbial communities through microbiome analysis. Additionally, MEVs help monitor disease progression and play key roles in systemic diseases. However, the use of MEVs as disease markers also faces challenges including their heterogeneity, difficult separation due to the lack of specific surface markers, the need for deeper characterization to understand their interactions with host cells, and standardization and optimization issues that must be overcome before clinical application^[144]^. Furthermore, issues related to safety and biocompatibility, especially toxins that MEVs may carry, and the lack of clinical trial data, also require further research and resolution. Despite these challenges, the potential of MEVs in disease diagnosis and treatment remains enormous, awaiting full exploration and utilization through future research and technological developments.

### Disease treatment

#### Bone

In the field of regenerative medicine, MEVs are also currently used for bone regeneration, demonstrating considerable therapeutic potential. MEVs can influence cellular processes^[145]^ and can be engineered to express specific targeting molecules on their surfaces through bioengineering strategies. This enables precise cell recognition and integration, thereby improving therapeutic effects and reducing side effects^[146]^. Additionally, these MEVs have high loading capacity and can carry growth factors, signaling molecules, or genetic material to stimulate osteoblast proliferation, differentiation and mineralization^[147]^.

Recombinant probiotics with bone-targeting and osteo inductive abilities were constructed, named MEVs-hCXCR4 (MEVs-C). SOST siRNA was integrated into these MEVs by electroporation to form MEVs-hCXCR4-SOST siRNA (MEVs-CSs), which effectively triggered the osteogenic differentiation of bone marrow mesenchymal stem cells (BMSCs) by regulating the WNT signaling pathway^[147]^. A novel engineered vesicle named Bone-Targeting *Lactobacillus rhamnosus* GG Extracellular Vesicle (BT-LGG-EV), utilizing bone-targeting peptides and EVs derived from probiotic LGG was developed. *In vitro* experiments showed that BT-LGG-EV could increase the formation of mineralized nodules and significantly enhance the expression of osteogenic genes *OCN, OSX, OPN, and Runx2*. TRAP staining and qPCR analysis indicated that BT-LGG-EV could inhibit osteoclast activity. In an ovariectomized (OVX) mouse model, BT-LGG-EV exhibited strong bone-targeting ability, promoting bone formation and improving osteoporosis by delivering miRNA^[148]^.

Jansen *et al.* evaluated the strengths and weaknesses of PCR, NGS, cultivation and FISH. They found that the human gut microbiome is a complex ecosystem composed of diverse microorganisms that play a crucial role in maintaining human health. Furthermore, disruptions in its composition are closely linked to a variety of health problems^[149]^. Another study discovered that colonization with the child gut microbiota (CGM), rather than the elderly gut microbiota, could prevent bone loss and the reduction of bone strength in ovariectomized osteoporosis mice. EVs derived from the CGM protected against osteoporosis by targeting *Akkermansia muciniphila* (Akk), enhancing osteogenic activity and inhibiting osteoclast formation^[150]^. Furthermore, Wang *et al.* demonstrated that OMVs of Gram-negative bacterium *Providencia* (P.M.) have the ability to inhibit osteoclast formation and bone resorption. These OMVs alleviated bone loss in experimental osteoporosis and rheumatoid arthritis by downregulating miR-96-5p^[151]^. These findings provide new approaches for developing bone regeneration strategies using bacteria.

In summary, MEVs show significant therapeutic potential in the field of bone regeneration. They effectively promote osteoblast proliferation and differentiation through precise cell recognition and loading of bioactive molecules. However, challenges remain in scale-up production, standardization, and clinical translation.

#### Skin

In the field of dermatopathology, MEVs have shown great promise in regulating skin immune responses, promoting wound healing, and preventing scar formation. The applicability and effectiveness of *Lactobacillus druckerii*-derived extracellular vesicles (LDEVs) in treating hypertrophic scars (HS) were reported by Han *et al.*^[152]^. *In vitro* experiments and animal models showed that LDEVs significantly inhibited the expression of collagen and α-smooth muscle actin (α-SMA) in fibroblasts, reduced cell proliferation, and promoted skin cell proliferation, neovascularization, and wound healing, thereby reducing the formation of hypertrophic scars.

Extracellular vesicles (SEStaphylococcus epidermidis mitigate inflammatory responses in a mouse model of atopic dermatitis (AD) by reducing proinflammatory gene expression, enhancing skin barrier function, and promoting keratinocyte proliferation and migration^[153]^. Additionally, SE-EVs increase the expression of human β-defensin and enhance resistance to *Staphylococcus aureus* by activating the Toll-like receptor 2 (TLR2) pathway. In the AD mouse model, application of SE-EVs was observed to significantly reduce inflammatory cell infiltration, TH2 cytokine gene expression, and IgE levels, indicating that SE-EVs may serve as effective bioactive nanocarriers for treating this condition. These findings provide a scientific basis for using EVs from commensal skin microbiota as a novel therapeutic strategy.

Chen *et al.* revealed that membrane vesicles (RMVs) secreted by *Lactobacillus reuteri* induce macrophages ploraization towards an anti-inflammatory state, enhancing mucosal and skin wound healing^[154]^. The study showed that RMVs could reduce the number of proinflammatory macrophages in inflamed tissues and regulate mitochondrial permeability of macrophages through their internal 3-hydroxypropionaldehyde (3-HPA) component. This led to reduced oxidative stress, prompting macrophage phenotypic transformation toward an anti-inflammatory state, which is conducive to wound healing. The potential of RMVs to promote wound healing was confirmed in both *in vitro* and *in vivo* experiments, providing a new method for using probiotic EVs to treat skin injuries.

Collectively, these studies indicate that MEVs hold great potential as innovative and clinically applicable therapies for skin diseases. By regulating host immune responses and promoting wound healing, MEVs offer new treatment approaches in dermatopathology and are expected to play a key role in future therapeutic strategies.

#### Others

MEVs have attracted extensive attention due to their potential therapeutic applications in various diseases. Preclinical studies have shown their abilities to promote placental development, alleviate preeclampsia, regulate biofilm formation, reduce stress-induced changes in the brain, and improve depressive behavior^[144,155]^. Notably, MEVs derived from probiotics were found to induce the expression of neurotrophic factors in the hippocampus, thereby countering stress-induced neuronal dysfunction^[156]^. Additionally, MEVs derived from *Lactococcus lactis* have been shown to stimulate IL-12 production, activate dendritic cells, and alter immune responses in allergic asthma, thereby reducing airway inflammation^[157]^. However, the clinical translation of MEVs requires in-depth understanding of their safety and the establishment of standardized regulatory frameworks. Further research is crucial for optimizing BEV production, purification, and characterization, with a focus on improving their delivery efficiency to targets.

Exploring various delivery systems, including hydrogels, scaffolds, and nanoparticles, may enhance the stability, targeting, and controlled release of MEVs. Ultimately, long term safety and efficacy studies are essential for establishing the role of MEVs in regenerative medicine treatments. Wu *et al.* proposed a method for supramolecular covalent cascade modification of cell membranes, through which biomimetic hydrogel materials (SFSHs) that mimic skin structure and function can be prepared^[158]^. This method uses bacterial outer membrane vesicles as crosslinking surfaces. Compared with traditional hydrogels formed by small molecule crosslinkers, SFSHs have several advantages. The deformation of vesicles can dissipate energy, thereby greatly improving the mechanical strength of SFSHs. Additionally, MEVs carry many bioactive substances derived from bacteria, which have unique inhibitory effects on pathogenic bacteria and can promote dendritic cell maturation. This study provides ideas for developing biomimetic skin materials with tunable structural and functional properties and exemplifies the feasibility of integrating MEVs with materials, including hydrogels for tissue engineering applications.

In summary, selecting biomaterials for loading MEVs is crucial. In recent studies, polyhydroxyalkanoates (PHA) have been widely used in tissue regeneration research due to their favorable biocompatibility and biodegradability. Similarly, it was reported that PHA can be used to load and deliver MEVs, which is a promising approach for developing advanced therapeutic strategies^[159,160]^.

### Medical cosmetology

In the field of medical cosmetology, EVs have gradually emerged. Stem cell-derived EVs can promote skin cell proliferation and collagen synthesis, improve skin texture, and reduce wrinkle formation. Studies have shown that mesenchymal stem cell derived EVs can stimulate fibroblasts to secrete more collagen and elastic fibers, increasing skin elasticity and firmness. Meanwhile, EVs also have anti-inflammatory properties, which can reduce skin inflammatory responses and improve sensitive skin conditions. For example, preclinical studies have applied EVs rich in growth factors to skin aging models, showing significant reduction in wrinkle depth and remarkable improvement in skin gloss and elasticity^[161]^.

MEVs derived from *Lactobacillus* have emerged as a core technology reported in recent review. Compared with skincare products derived from mesenchymal stem cells or plants, microbial EV-based skincare products are less popular. Given that most EVs from skin microbiota exert beneficial skincare effects, they possess significant market potential for developing more cosmetics based on EVs derived from single or multiple bacterial strains^[130]^.

Kang *et al.* reported that Lactobacillus-derived artificial extracellular vesicles (LAEs) effectively promote wound healing in fibroblasts and regulate ageing-related genes, making them potential alternative to natural EVs for skin rejuvenation and anti-aging applications^[162]^.

### Functional foods

In the field of functional foods, EVs have also begun to attract attention. EVs derived from certain probiotics have been confirmed to regulate the intestinal microecological balance and enhance intestinal barrier function, allowing them to be added to foods as functional ingredients. For example, a study published in the *Food Bioscience* in 2025 pointed out that EVs produced by *Lactobacillus plantarum* can modulate macrophage polarization and regulate gut homeostasis, thereby alleviating ulcerative colitis and improving the intestinal environment. It has been proposed that utilizing these *Lactiplantibacillus plantarum*-derived EVs as a combinatorial therapy with 5-ASA is expected to reduce drug dosage requirements and serve as a highly promising nanoscale therapeutic alternative for treating gastrointestinal inflammation^[163]^. Additionally, EVs may serve as delivery carriers for nutrients, improving their bioavailability. For example, engineering EVs to load nutrients such as vitamins and minerals can promote their absorption in the intestine. However, research in this field is still in its infancy. Consequently, comprehensive exploration remains necessary concerning EV safety assessment, large scale preparation, and application stability.

## CONCLUSION

Both Gram-positive (G+) and Gram-negative (G-) bacteria can produce MEVs. Gram-negative bacteria can produce OMVs, inner-outer membrane vesicles (OIMVs), and explosive outer membrane vesicles (EOMVs). Gram-positive bacteria can produce cytoplasmic membrane vesicles (CMVs). The cargo of MEVs produced by G+ and G- bacteria differ not only in the presence or absence of lipopolysaccharide (LPS) but also in other molecules such as nucleic acids, proteins, lipids, and metabolites^[164-166]^. The isolation and purification of MEVs are crucial for their subsequent applications, but currently, there is a lack of standardized isolation and purification methods. This article summarizes common MEVs isolation and purification protocols.

MEVs have been developed as novel drug delivery platforms. With the wide application of gene editing technologies in bacteria, knocking out certain genes or expressing foreign genes can affect the secretion of EVs and their cargo, endowing MEVs with great potential in drug delivery applications. Since MEVs can carry a variety of antigens, they are applied in vaccine development. Additionally, MEVs can reflect the status and function of source bacteria, so they are valuable disease diagnostic markers. In the medical field, MEVs show considerable therapeutic potential in bone regeneration and also play important roles in the treatment of other diseases. MEVs exhibit characteristics such as efficient drug delivery, ease of industrialization, and convenient modification, while also being applicable for the diagnosis of bacterial infections.

Despite the remarkable potential of MEVs in drug delivery, vaccinology, diagnostics, and therapy, their translation from promising research tools into clinically approved products faces a series of significant hurdles. A major barrier is the lack of a unified global regulatory framework and standardized characterization protocols for identity, purity, and potency, impeding product comparability and approval. Scalable manufacturing under Good Manufacturing Practice (GMP) conditions remains a core bottleneck, with current methods struggling to balance yield, cost, and vesicle integrity. MEV instability (prone to membrane fusion and cargo leakage) limits commercialization, and current cryopreservation hinders widespread adoption. Biosafety concerns include LPS in Gram-negative MEVs, immunogenicity of engineered MEVs, and off-target risks, with ethical debates over gene-edited chassis^[127]^.

MEVs as delivery systems or therapeutic drugs still face many challenges in clinical translation, including potential biosafety issues, complex and time-consuming separation procedures, and poorly characterize cargo with unclear mechanisms of action. Future advancement hinges on interdisciplinary collaboration to establish regulatory standards and develop robust, cost-effective GMP production processes. By integrating synthetic biology for design and nanotechnology for function, MEVs are poised to evolve from a promising research tool into a transformative platform in nanomedicine for therapeutic and diagnostic applications.

## DECLARATIONS

### Copyright

© The Author(s) 2026.
